# Development a hyaluronic acid ion-pairing liposomal nanoparticle for enhancing anti-glioma efficacy by modulating glioma microenvironment

**DOI:** 10.1080/10717544.2018.1431979

**Published:** 2018-01-29

**Authors:** Liuqing Yang, Xu Song, Ting Gong, Kejun Jiang, Yingying Hou, Tijia Chen, Xun Sun, Zhirong Zhang, Tao Gong

**Affiliations:** Key Laboratory of Drug Targeting and Drug Delivery Systems, Ministry of Education, West China School of Pharmacy, Sichuan University, Chengdu, PR China

**Keywords:** Hyaluronic acid, glioma, TAMs, CD44, CSCs

## Abstract

Glioma, one of the most common brain tumors, remains a challenge worldwide. Due to the specific biological barriers such as blood–brain barrier (BBB), cancer stem cells (CSCs), tumor associated macrophages (TAMs), and vasculogenic mimicry channels (VMs), a novel versatile targeting delivery for anti-glioma is in urgent need. Here, we designed a hyaluronic acid (HA) ion-pairing nanoparticle. Then, these nanoparticles were encapsulated in liposomes, termed as DOX-HA-LPs, which showed near-spherical morphology with an average size of 155.8 nm and uniform distribution (PDI = 0.155). HA was proven to specifically bind to CD44 receptor, which is over-expressed on the surface of tumor cells, other associated cells (such as CSCs and TAMs) and VMs. We systematically investigated anti-glioma efficacy and mechanisms *in vivo* and *in vitro*. The strong anti-glioma efficacy could attribute to the accumulation in glioma site and the regulation of tumor microenvironment with depletion of TAMs, inhibition of VMs, and elimination of CSCs.

## Introduction

Malignant glioma, with high mortality and morbidity, has been one of the most aggressive and deadly tumors (Shi et al., 2015). Due to the difficulty in surgical resection of glioma, radio- and chemotherapy have played an important role in glioma treatment. However, radiotherapy failed to improve prognostic (Ruan et al., 2016). Chemotherapy, as a commonly used treatment agent, still has several obstacles. BBB (blood–brain barrier) could lead to systemic toxicity and poor drug penetration which attributed to Cohen et al. (2015). Besides, brain cancer stem cells (brain CSCs) played an important role in tumor initiation, propagation, metastasis, and recurrence (Vescovi et al., 2006). Vasculogenic mimicry channels (VMs) provided blood supply for invasion, promoted tumor growth and exhibited high resistance to conventional chemotherapy (Zhang et al., 2007). What is more, tumor associated macrophages (TAMs) contributed to tumor growth, angiogenesis, metastasis, immunosuppression, matrix deposition, and remodeling (Hao et al., 2012). Thus, developing a novel versatile drug delivery system (DDS) is in great demand. Recently, nanodrug delivery systems have attracted much attention because of the optimized size, zeta-potential, and specific receptor mediated tumor-targeting ligands (Shao et al., 2014).

Hyaluronan (HA), a naturally anionic cell-surface-associated polysaccharide, is an important component of the extracellular matrix showing a crucial role in cell growth, tissue remodeling, inflammation, and tumor progression (Cohen et al., 2015). HA was proven to specifically bind to CD44 receptor which is over-expressed on the surface of several cancer cells for the development of tumor cell targeting delivery system (Song et al., 2014). Hayward et al. modified liposomes by HA chemical conjugated (HALNPs) for U87-GBM targeting (Hayward et al., 2016). Cohen et al. devised an *in situ* strategy to deliver RNAi directly to U87-GBM site using HA-grafted lipid-based nanoparticles (LNPs) (Cohen et al., 2015). However, treatment aiming at tumor microenvironment (TME) has caught public attention.

Microenvironmental cells such as TAMs and CSCs play an important role in tumor growth. Due to their role in angiogenesis, metastasis, and recurrence, selective targeting might prove to be a valuable strategy for tumor treatment. In a previous study, Shi et al. developed paclitaxel-loaded liposomes by conjugating with a TR peptide as an integrin αvβ3-specific vector for transporting drug into glioma against CSCs (Shi et al., 2015). Song et al. utilized an HA-grafted MnO_2_ nanoparticle drug delivery for reducing M_2_ polarized macrophages in breast cancer (Song et al., 2016). Those therapies demonstrated inhibiting CSCs or TAMs could achieve a desired antitumor effect. Up to now, it has been reported that TAMs and CSCs were shown to overexpress CD44 receptors (Shi et al., 2015; Huang et al., 2016). Furthermore, HA was also identified as a novel marker of VMs showing an important function for CD44 in the formation of vascular-like tumor cell networks (Paulis et al., 2015). As aforementioned, HA was widely used as specific ligands for CD44 highly expressed tumor cells, which lead to a stronger anti-tumor efficacy. Based on these, we hypothesized that CD44, overexpressed on TAMs and CSCs, as well as VMs, could be used as the target site for anti-glioma requirements.

**Figure 1.**
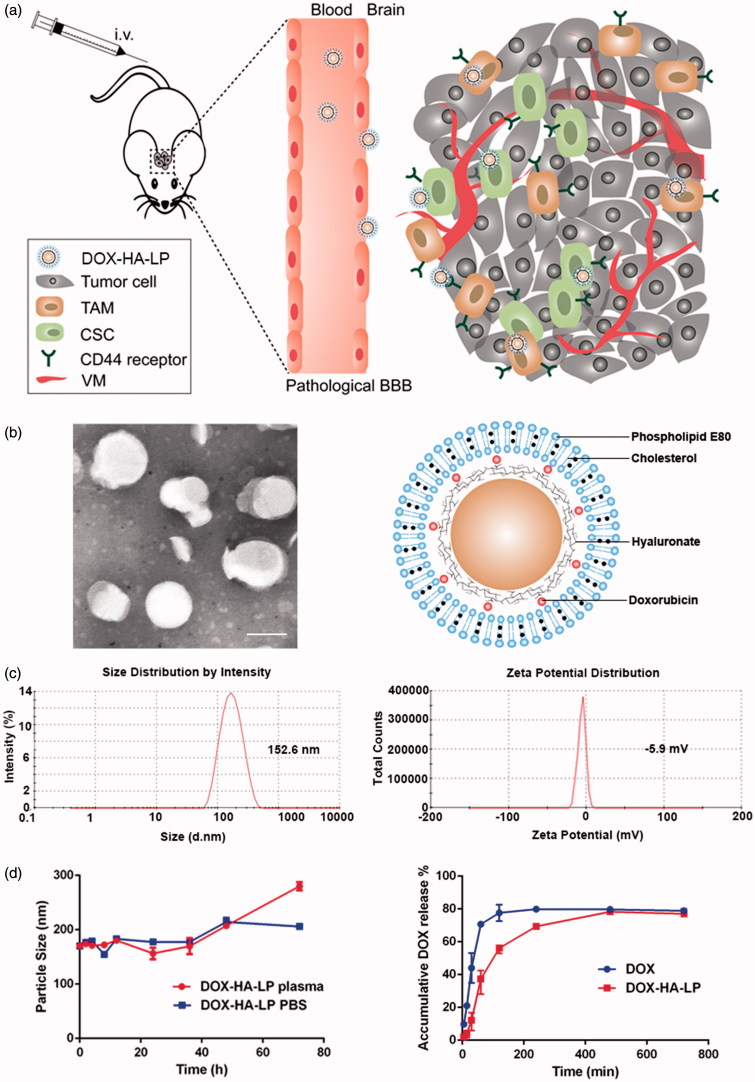
Characterization of DOX-HA-LPs. (a) Schematic illustration of DOX-HA-LPs, which is transported into glioma site after tail vein injection, targeting TAMs and CSCs mediated by CD44, resulting in depletion of TAMs, inhibition of VMs and elimination of CSCs; (b) TEM image and schematic diagrams of DOX-HA-LPs. Scale bar, 100 nm; (c) DLS size and zeta-potential measurement of DOX-HA-LPs; (d) the stability of DOX formulation in plasma and PBS for 72 h; DOX release profiles of DOX formulations at pH 7.4 (*n* = 3, mean ± SD).

In our previous study, we have designed a hyaluronic acid (HA) ion-pairing nanoparticle via ion-pairing between positively charged doxorubicin (DOX) and negatively charged HA, termed as HA-LPs. Maintaining the initial structure of HA without any chemical modification, HA-LPs showed a high accumulation ability in tumor site and a strong anti-tumor efficacy against H22 (murine hepatoma cells, which expressed CD44 highly) (Li et al., 2016). Now, on the basis of the above background, we investigated the influence of DOX-HA-LPs on glioma and TME (Figure 1(a)). We employed murine glioma C6 cells to systematically investigate cellular uptake and VMs inhibiting capability *in vitro*. Meanwhile, the anti-glioma efficacy and mechanisms were further demonstrated by mice bearing C6 glioma.

## Methods

### Materials

Sodium hyaluronate (HA, 77 kDa) was obtained from Freda Biopharm Co. Ltd. (Shangdong, China). Doxorubicin hydrochloride was obtained from Beijing Huafeng United Technology Co. Ltd. (Beijing, China). Phospholipid E80 (E80) was purchased from Lipoid Co. Ltd. (Ludwigshafen, Germany). Cholesterol (Chol) was obtained from Kelong Company (Chengdu, China). Solutol-HS 15 was thankfully obtained from BASF Aktiengesellschaft (Ludwigshafen, Germany) and soybean oil (for injection) was purchased from Tieling Medical Oil Co. Ltd. (Tieling, China). Interleukin-4 mouse recombinant (IL-4) was purchased from R&D (Minneapolis, MN). Lipopolysaccharides (LPS) and DAPI dye were purchased from Sigma-Aldrich (St. Louis, MO). Anti-Mannose receptor antibody [15-2], periodic acid Schiff (PAS) and anti-CD31 staining kit were purchased from Abcam Company (Cambridge, UK). Other chemicals and reagents were analytical grade or better.

### Animals

Male BALB/c mice (18–22 g) and male SD rats (180–200 g) were purchased from Chengdu Dashuo Experiment Animal Center (Chengdu, China). All animals in this study were performed according to the guidelines from Sichuan University and with approval of the Ethics Committee of Sichuan University.

### Preparation and characterization of DOX-HA-LPs

DOX-HA-LPs were prepared mainly as previously described (Li et al., 2016). Briefly, HA, HS-15, and soybean oil for injection in mass ratio of 1:5:1 were dissolved in deionized water, then the mixture aforementioned was stirred for 15 min at room temperature to get white turbid liquid. We mixed the white turbid liquid with deionized water containing DOX (HA-to-DOX feeding ratio was molar ratio 2:1) for 30 min reaction at room temperature. Next, the mixture obtained above was homogenized (AH100D, ATS Engineering Inc. Nano Homogenize Machine, Brampton, Canada). Finally, we use thin-film hydration method to capsulated DOX-HA-NPs into liposomes. Briefly, E80 and Chol were dissolved in dichloromethane at mass ratio of 2:1 and the organic solvent was removed by rotary evaporation at 37 °C to form a thin phospholipid film. Then, the film was hydrated by 10 mL of DOX-HA-NPs suspension at 37 °C, and further homogenized.

The particle size distribution and zeta potentials of DOX-HA-LPs were determined by dynamic light scattering (DLS) (Malvern Zetasizer Nano ZS90, Malvern, UK) at 25 °C. The size and morphology of DOX-HA-LPs were observed by transmission electron microscope (TEM, H-600, Hitachi, Japan) following negative staining with 2% phosphotungstic acid.

The entrapment efficiency (EE) of DOX was determined by ultracentrifugation method. Briefly, 0.5 mL DOX-HA-LPs were destroyed with 1% HCl ethanol and measured by fluorescence spectrophotometer (RF5301 PC, Shimadzu, Kyoto, Japan) at the wavelength (*E*_x_/*E*_m_) of 498/584 nm to determine the total amount of DOX (*W*_t_). In addition, another 0.5 mL DOX-HA-LPs were ultracentrifuged at 40,000 rpm for 30 min (Optima MAX-XP, Beckman Coulter, Brea, CA) and the supernatant was collected for determining the free DOX (*W*_f_) in formulations. Therefore, EE could be calculated according to the following equation: EE (%) = (*W*_t_ – *W*_f_)/*W*_t_×100%.

The colloidal stability of DOX-HA-LPs in PBS (pH 7.4) or rat plasma was evaluated by measuring the changes in particle size. Briefly, DOX-HA-LPs were mixed with equal volume of plasma or PBS at 37 °C with shaking of 30 rpm. At time points, 100 μL of sample was diluted to 1 mL with deionized water for particle size measurement by DLS.

*In vitro* release of DOX from DOX-HA-LPs was investigated by dialysis. Briefly, 0.5 mL different DOX formulations were added into dialysis bags (MWCO = 3500 Da) and immersed into 40 mL of release medium (PBS, 0.01 M, pH 7.4) at 37 °C with shaking of 100 rpm for 12 h. At predetermined time points, 1 mL of medium was collected and replenished with an equal volume of fresh one.

### Cell cultures

Murine glioma (C6) and renal tubular epithelial cells (HK2, no expression of CD44) were cultured in a humidified 5% CO_2_ atmosphere at 37 °C. HK2 was cultured in RPMI-1640 medium and C6 was cultured in Dulbecco’s modified Eagle’s medium (DMEM). Both of the medium was supplemented with 10% FBS (Hyclone, Logan City, UT), 100 U/mL penicillin and streptomycin (Solarbio, Beijing, China).

Glioma C6 cancer stem like cells (C6 CSCs) were cultured as previously described (Shi et al., 2015). Briefly, it is cultured in serum-free DMEM (Gibco, Carlsbad, CA) supplemented with 20 ng/mL basic fibroblast growth factor (bFGF), 20 ng/mL epidermal growth factor (EGF) and 2% B27 (Gibco, Carlsbad, CA).

### Cellular uptake

To stimulate M_1_ or M_2_ polarization in macrophages, 50 ng/mL LPS or 20 ng/mL mouse interleukin 4 (IL-4) was added to RAW 264.7 cells, and the resulting mixture incubated for 96 h at 37 °C (Liu et al., 2014). Polarized and control RAW 264.7 cells were seeded in 12-well plates at 1 × 10^5^ cells per well and incubated for 12 h. Then, 5 μg/mL of free DOX, DOX-HA-LPs were added to culture medium. After 4 h, cells were collected for analysis by flow cytometer (Cytomics^TM^ FC 500, Beckman Coulter, Brea, CA).

To demonstrate targeting ability of DOX-HA-LPs, C6, HK2 cells were seeded in 12-well cell culture plates at 1 × 10^5^ cells per well and incubated for 12 h. As aforementioned, after 4 h incubation with different DOX formulations, cells were collected for analysis by flow cytometer. Therein, HK2 cells served as a negative control cell line with no expression of CD44 receptor. Moreover, uptake mechanism was investigated with pretreatment of free HA (10 mg/mL) (Li et al., 2016).

### Endocytosis pathways

To explore the endocytosis pathway of DOX-HA-LPs on glioma C6 cells, endocytosis inhibition assay was employed (Shi et al., 2015). Briefly, C6 cells were seeded in 12-well cell culture plates at 1 × 10^5^ cells per well and incubated for 12 h. Then, cells were preincubated with inhibitors (free HA, amiloride, β-CD, verapamil, chlorpromazine, nystatin, and N_3_Na) for 1 h at 37 °C, respectively, before another incubation with 5 μg/mL of DOX-HA-LPs for 4 h. The cells were collected for analysis by flow cytometer.

### Cytotoxicity assay

Cellular cytotoxicity of free DOX, DOX-HA-LPs, DOX-HA^(−)^-LPs (the same preparation method with DOX-HA-LPs, only without HA) and blank liposomal nanoparticles (the same preparation method with DOX-HA-LPs, only without DOX) was assessed by MTT assay. Glioma C6 cells were seeded in 96-well plates at 1 × 10^4^ cells per well and incubated in a humidified 5% CO_2_ atmosphere at 37 °C for 12 h. Then, the medium was replaced with serum-free culture medium containing varying concentrations of free DOX, DOX-HA-LPs, DOX-HA^(−)^-LPs and blank liposomal nanoparticles (HA concentration as a standard). After 24 h incubation, MTT solution (5 mg/mL in PBS) was added and further incubated for 4 h under 37 °C. Finally, medium was discarded and DMSO was added to dissolve the formazan for analysis by microplate reader (Thermo Scientific Varioskan Flash, Waltham, MA) at 490 nm. Cell viability (%) = *A*_test_/*A*_control_ × 100%, where *A*_test_ and *A*_control_ represented the absorbance of treated cells and blank cells, respectively.

### Inhibition against glioma VMs

A Matrigel-based tube formation assay was used to assess the inhibitory effect against VMs. Briefly, 200 μL of Matrigel (BD Biosciences, Franklin Lakes, NJ) was plated onto 24-well cell plates, and incubated for 30 min until solidification. C6 cells (1 × 10^4^) were resuspended with serum-free medium containing free DOX, DOX-HA-LPs, and free HA, and seeded onto the Matrigel. The final concentration of DOX was 5 μg/mL. The content of HA in free HA was the same with that in DOX-HA-LPs. After incubation at 37 °C for 1 h and 4 h, the state of VMs was analyzed by Carl Zeiss Jena Axiovert 40 inverted microscope (Oberkochen, Germany).

### Pharmacokinetic study

Male SD rats (200 ± 20 g) were divided into two groups, randomly (*n* = 5). Rats were fasted overnight with free access to water before experiment. Free DOX and DOX-HA-LPs were administered intravenously with a dose of 5 mg/kg. At predetermined time points (2, 10, 20, 30, 60, 120, 240, and 360 min), blood was collected intraorbitally. One hundred microliters of plasma samples were collected following centrifugation, for analysis by LC–MS (Agilent 6410, Agilent Technologies, Palo Alto, CA). Drug and Statistics Software (DAS 2.0, Mathematical Pharmacology Professional Committee of China, Shanghai, China) was used to analyze the measured plasma concentration data of DOX.

### Glioma model establishment and biodistribution

Balb/c mice were anesthetized and fixed into a stereotactic device. C6 (1 × 10^6^ cells/5 μL) was injected into the right striatum (Li et al., 2014). After 10 days, glioma-bearing mice were divided into two groups, randomly (*n* = 20) and injected intravenously with free DOX and DOX-HA-LPs at a dose of DOX 5 mg/kg. The mice were sacrificed at 0.5 h, 1 h, 2 h, and 4 h after injection. Vital organs (heart, liver, spleen, lung, and kidney) and glioma-bearing brain were excised after heart perfusion with saline for *ex vivo* imaging by Caliper IVIS Lumina III (PerkinElmer, Waltham, MA). Glioma-bearing mice injected intravenously with saline served as a negative control.

After collecting *ex vivo* imaging, the organs (*n* = 5) were weighed and homogenized with two-folds of physiological saline (g/mL). The concentrations of DOX in different organs were determined by LC–MS.

For further study of the targeting ability to TAMs of DOX-HA-LPs, brain samples were fixed in 4% paraformaldehyde and dehydrated with sucrose, cut cryosections at a thickness of 10 mm and stained for TAMs with specific CD206 antibody (Luo et al., 2006). Cell nuclei were stained by DAPI. Sections were visualized by confocal laser scanning microscopy (CLSM) (A1R MP+, Nikon, Tokyo, Japan).

### Anticancer efficacy

#### Survival of glioma-bearing mice

Glioma-bearing mice were divided into three groups (*n* = 12), At 5, 8, 11, and 14 days after implantation, mice were injected intravenously with saline, free DOX, DOX-HA-LPs at a dose of 2 mg/kg. At the 15th day, two mice from each group were sacrificed for immunohistochemical analysis (H&E staining), the rest were used for the survival curves. Kaplan–Meier’s survival curves were plotted for each group.

*Glioma VMs*. To demonstrate the destruction of different DOX formulations against glioma VMs *in vivo*, periodic acid-Schiff (PAS) and anti-CD31 dual staining was used for VMs monitoring (Liu et al., 2015).

*Brain CSCs*. CD133 is a marker of CSCs in glioma (Wang et al., 2015). Brain sections of sacrificed glioma-bearing mice were stained with the method of immunoperoxidase for CD133. Reactions were visualized using horseradish peroxidase. Besides, cell nuclei were counterstained using hematoxylin, dehydrated and sealed.

*TAMs*. CD206 is a marker of TAMs in tumor (Luo et al., 2006). Cut paraffin sections at a thickness of 10 mm and stained for TAMs with specific CD206 antibody. Cell nuclei were stained by DAPI.

### Safety evaluation

Healthy male balb/c mice were randomly divided into three groups (*n* = 7), and administrated intravenously with saline, free DOX, and DOX-HA-LPs at a dose of 5 mg/kg. After three days of injection, blood samples were collected intraorbitally in Eppendorf tubes (containing EDTA – 2K^+^), and analyzed by auto hematology analyzer (BC – 2800 Vet, Mindray, Shenzhen, China) for platelets (Plt), white blood cells (WBCs), and red blood cells (RBCs).

Healthy male balb/c mice were randomly divided into three groups (*n* = 3), and administrated intravenously with saline, free DOX and DOX-HA-LPs at a dose of 2 mg/kg every three days for four times. At 13th day, mice were sacrificed. Heart and brain were excised after heart perfusion with saline and prepared for histological analysis by H&E staining.

### Statistical analysis

All assays were repeated three times at least. All data were represented as mean ± standard deviations (SD). Student's *t*-test and one-way ANOVA were performed for comparison between two and three groups, respectively. *p* Value <.05 was considered statistically significant.

## Results

### Preparation and characterization of DOX-HA-LPs

For maintaining the initial structure of HA without any chemical modification, we use attractive interactions between positive and negative charge to form nanoparticles. DOX-HA-LPs showed near-spherical morphology (Figure 1(b)) with an average size of 155.8 nm, a uniform distribution (PDI = 0.155) and a negative zeta potential of −5.9 mV (Figure 1(c)). EE (%) of DOX in DOX-HA-LPs was around 94.5% (Table S1).

High molecular weighted HA suspensions flocculated easily in a period time. Thus, evaluation of particle stability against physiological condition (50% plasma or PBS was employed to mimic physiological condition) would be a prerequisite for the further application *in vivo*. As shown in Figure 1(d), particle size was stable both in plasma and PBS over 36 h, indicating that there was no aggregation in plasma or PBS. But the particle size of DOX-HA-LPs increased 50 nm approximately both in plasma and PBS after 36 h, then increased about 100 nm after 72 h in plasma.

As shown in Figure 1(d), compared with rapid release of free DOX (approximately 90% of drug released into the medium within 2 h), DOX-HA-LPs exhibited slightly sustained release manner with approximately 90% of drug released into the medium up to 6 h.

### HA selectively targets to CD44^+^ cells

**Figure 2.**
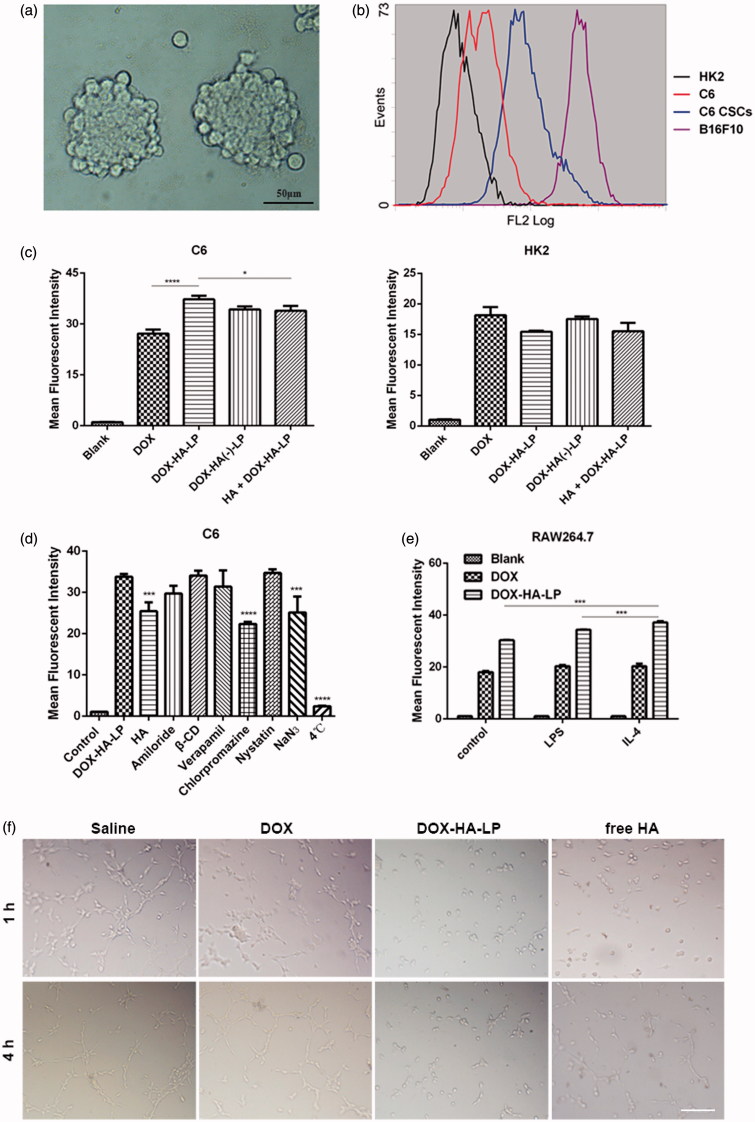
The effect of different formulations on cells. (a) Microscopic image of C6 CSCs spheres. Scale bar, 50 μm; (b) expression of CD44 at C6, C6 CSCs, B16F10 (as a positive control cell line) and HK2 (as a negative control cell line) cells; (c) cellular uptake of DOX formulations after incubation with HK2, C6 cells for 4 h. HA + DOX-HA: cells were incubated with HA solution (10 mg/mL, 100 kDa) in advance. Data represent mean ± SD (*n* = 3). *p < .05, **p < .01, ****p < .0001; (d) endocytosis inhibition assay on C6 cells. Data represent mean ± SD (*n* = 3). ***p < .001. ****p < .0001; (e) DOX-HA-LPs selectively targeted IL-4 treated RAW 264.7. Data represent mean ± SD (*n* = 3). ***p < .001; (f) the destruction of VMs channels after treatment with different DOX formulations. Scale bar, 100 μm.

We cultured glioma C6 CSCs (Figure 2(a)) as previously described and demonstrated the high expression of CD44 in CSCs (Figure 2(b)). HA was proven to specifically bind to surface CD44 receptor which is over-expressed on cancer cells, CSCs or other associated cells (such as TAMs and CAFs). HK2 cells (as a negative control cell line) and C6 cells were chosen to investigate the targeting property of HA. Besides, pretreatment with 10 mg/mL HA (100 kDa) solution was used to elucidate the uptake mechanism. As shown in Figure 2(c), fluorescence intensity of DOX-HA-LPs was 1.4-fold higher than that of free DOX on C6. It was also seen no significant difference on HK2 cells. Free HA macromolecules might compete for the CD44 binding sites. In HA pretreatment group, the fluorescence intensity of DOX-HA-LPs was reduced significantly on C6 cells. However, these changes were not shown on HK2 cells. All data showed that DOX-HA-LPs could be internalized into cells via CD44 receptor-mediated endocytosis.

### Endocytosis pathway

To elucidate cellular uptake mechanism of DOX-HA-LPs, different endocytosis inhibitors were evaluated on C6 cells. As shown in Figure 2(d), the cellular uptake of DOX-HA-LPs after treatment with chlorpromazine (a clathrin inhibitor), NaN_3_ (energy inhibitor), and incubation at 4 °C (energy inhibitor) decreased by 34% (*p* < .0001), 26% (*p* < .001), and 90% (*p* < .0001), respectively. Furthermore, consistent with the result of cellular uptake assay, fluorescent intensity of DOX-HA-LPs was decreased by 25% (*p* < .001) with the pretreatment of free HA solution, suggesting that clathrin-mediated, energy-depended, and CD44 receptor-mediated endocytosis had an important role in uptake of DOX-HA-LPs.

### DOX-HA-LPs induce cytotoxic effect on C6 and RAW 264.7 cells

All formulations showed dose dependent anti-tumor cell activity. The IC_50_ values of DOX-HA-LPs against C6 and RAW 264.7 cells were 6.6 ± 0.4 and 0.1 ± 0.007 μg/mL, respectively (Table S2). DOX-HA-LPs showed a significant anti-tumor cell property compared with free DOX (*p* < .0001). Besides, the IC_50_ values of blank liposomal nanoparticles and DOX-HA^(−)^-LPs against C6 cells were 4.1- and 2.2-fold higher than that of DOX-HA-LPs due to the absence of DOX and HA, respectively.

### DOX-HA-LPs inhibit the formation of VMs

VMs might help tumor cells to increase malignancy and obtain sufficient oxygen and nutrients (Zhang et al., 2007). Thus, demonstrating the inhibitory activity of DOX-HA-LPs against the VMs was necessary. As shown in Figure 2(f), after treatment with different formulations for 1 h and 4 h, C6 cells formed vessel-like loops, networks and channels in Matrigel. DOX-HA-LPs exhibited significantly stronger inhibitory property than free DOX and free HA. HA also showed better inhibitory potency than free DOX. As reported, in aggressive tumors, overexpression of CD44 might help tumor cells adhere to extracellular matrix and facilitate the formation of VMs (Paulis et al., 2015). Our findings suggested that DOX-HA-LPs and HA might inhibit VMs and tumor angiogenesis.

### DOX-HA-LPs exhibit a different circulating behavior and a glioma-bearing brains concentrated capability

The concentration–time curves of different DOX formulations are displayed in Figure S1. The clearance of free DOX was finished quickly within 3 h. In contrast, DOX-HA-LPs continued releasing for at least 6 h, with AUC_(0–∞)_ 2.5-fold higher than free DOX (Table S3).

**Figure 3.**
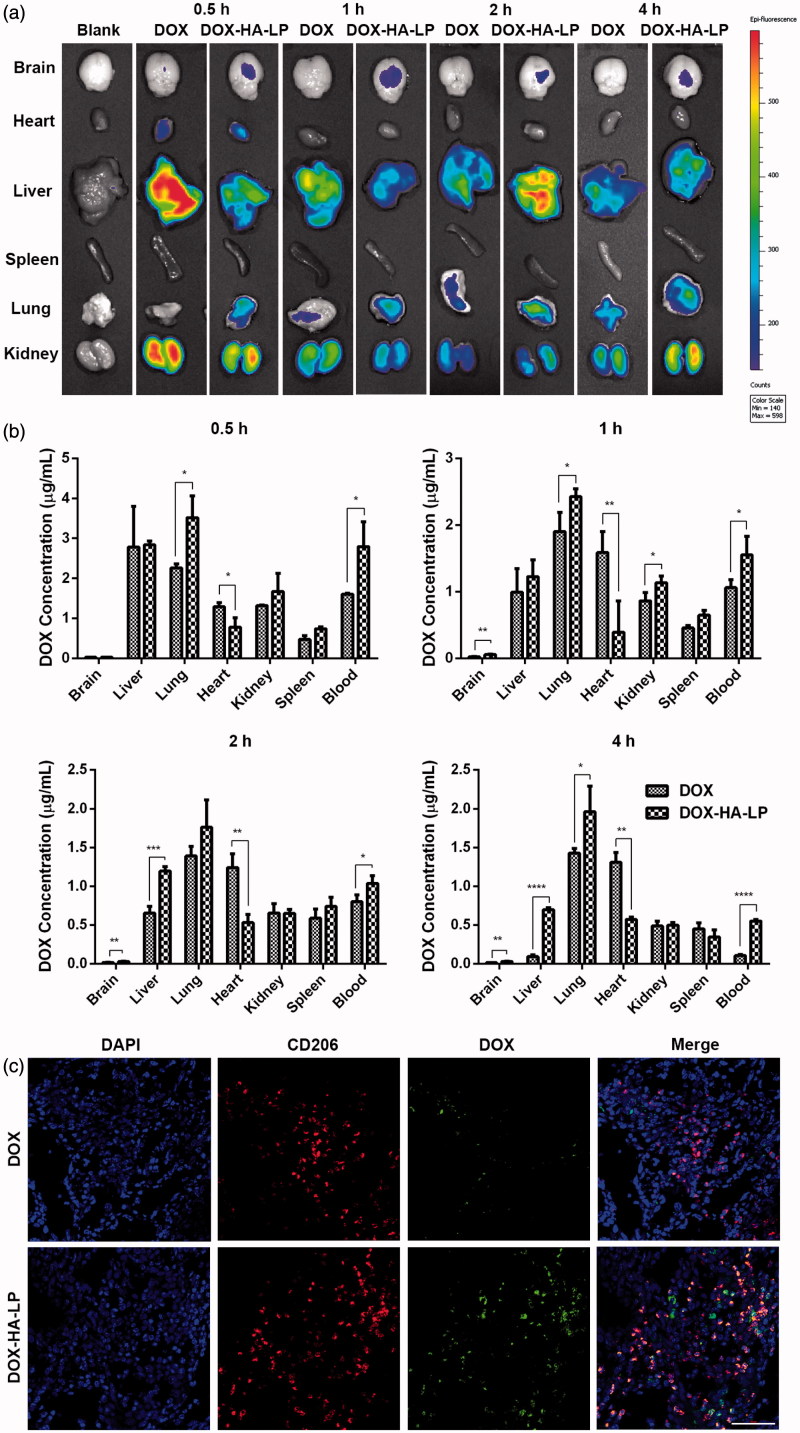
DOX-HA-LPs exhibit a different circulating behavior and a glioma-bearing brains concentrated capability. (a) *Ex vivo* images showed the drug biodistribution of DOX formulations in C6 glioma-bearing mice at 0.5, 1, 2, and 4 h after intravenous administration; (b) the mean concentrations of DOX in different tissues of C6 glioma-bearing mice after intravenous administration of DOX formulations at 0.5, 1, 2, and 4 h. Data represent mean ± SD (*n* = 5); (c) *in vivo* fluorescent microscopy images of brain sections from C6 glioma-bearing mice at 1 h post-injection of DOX and DOX-HA-LPs. TAMs were identified by CD206 marker and nuclei stained with DAPI. Scale bar, 50 μm.

To evaluate tumor targeting property of DOX-HA-LPs, glioma-bearing balb/c mice were employed. As shown in Figure 3(a,b), fluorescence signal of DOX-HA-LPs in glioma-bearing brains was stronger than free DOX at each predetermined time points, particularly at 1 h time point. In addition, fluorescence of DOX-HA-LPs in hearts was weaker than free DOX at each given time points, suggesting that DOX-HA-LPs might reduce the cardiac toxicity.

### DOX-HA-LPs selectively target TAMs

Influenced by TME cytokines, macrophages would be polarized to M_1_ and M_2_ macrophages. M_2_ polarization, induced by IL-4, takes an important role in tumor growth, development, invasion, and metastasis. As shown in Figure 2(e), the uptake of DOX-HA-LPs was significantly increased on M_2_ (*p* < .001) polarization, while there was no change in free DOX. *In vivo*, our data also demonstrated that DOX-HA-LPs achieved selective targeting to M_2_ macrophages compared to free DOX (Figure 3(c)).

### Anti-glioma efficacy *in vivo*

**Figure 4.**
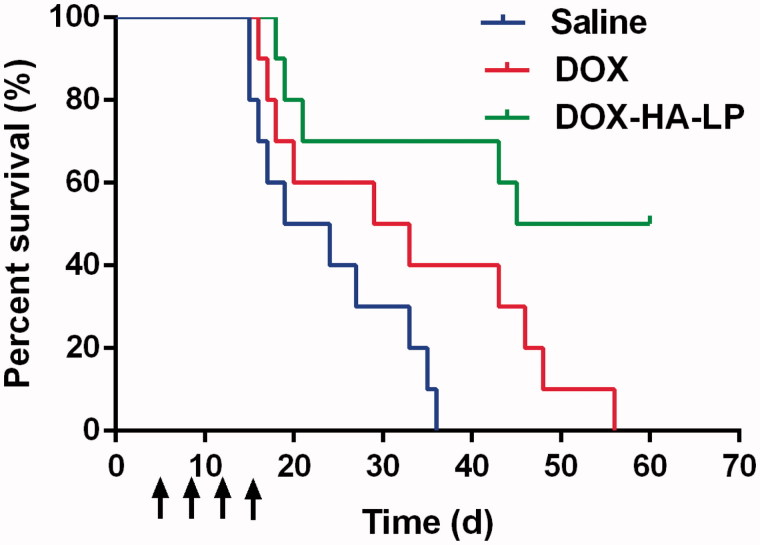
The survival curves of C6 glioma-bearing mice treated with DOX formulations (2 mg/kg DOX) at day 5, 8, 11, and 14 after inoculation. Data represent mean ± SD (*n* = 10).

**Figure 5.**
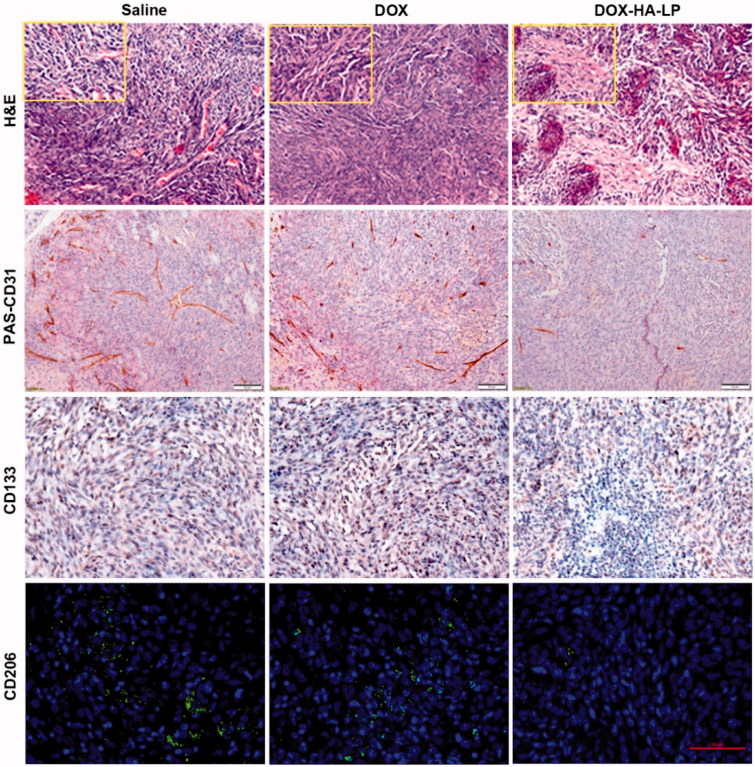
H&E staining of brain glioma site at 24 h after antitumor dosage administration of saline, free DOX and DOX-HA-LPs. Scale bar, 50 μm and 20 μm (small boxes). CD34-PAS dual staining, CD133 staining, CD206 staining on the brain glioma site. Scale bar, 50 μm.

Survival curves (Figure 4) indicated that DOX-HA-LPs showed the anti-glioma efficacy. In DOX-HA-LPs group, 50% of mice remained alive after 60 days with a median survival of 53 days (Table S4), which significantly prolonged compared with saline (21.5 days, *p* < .01) and free DOX (31 days, *p* < .05) group. H&E staining of tumor sites (Figure 5) demonstrated that *in vivo* DOX-HA-LPs group showed a significant suppression against proliferation of C6, while free DOX group displayed no significant improved anti-glioma efficacy compared with saline group. CD31-PAS dual staining was used to exhibit the destroying effect on VMs. DOX-HA-LPs showed a prominent destroying effect against VMs compared with free DOX. CD133 immunohistochemical staining on glioma sites is displayed in Figure 5. Based on the size of brain CSCs area, anti-CSCs efficacy of DOX-HA-LPs was stronger than free drug. TAMs were stained by CD206. DOX-HA-LPs could significantly deplete TAMs, compared with free DOX.

### DOX-HA-LPs show lower toxicity than free DOX

Bone marrow suppression and cardiac toxicity were the major side effects of DOX. To investigate suppression effect of bone marrow of different DOX formulations, several hematological parameters were measured on 3rd day after treatment. The appearance of bone marrow suppression and abnormality in immune system was analyzed by the counts of Plt, WBC, and RBC. As shown in Figure S2(a), treatment with free DOX induced a significant decrease in Plt (*p* < .05) and WBC (*p* < .01) compared with DOX-HA-LPs, while DOX-HA-LPs showed no significant difference with saline indicating lower bone marrow suppression. It has been reported that free DOX causes dose-limiting systemic toxicity, particularly cardiac toxicity in clinic (Kim et al., 2014). To research cardiac toxicity of different DOX formulations, histopathology observations were performed on the hearts in different groups using H&E staining. As shown in Figure S2(b), there was severe cardiac toxicity in free DOX group such as inflammation, myocardial cell atrophy and breakage. Only myocardial cell atrophy was rarely observed in DOX-HA-LPs group. As to brain, there was cerebral vasodilatation and screen-like cavity in DOX group, while no vasodilatation, hyperemia, necrosis, or inflammation in DOX-HA-LP group, suggesting that DOX-HA-LPs had a relatively low systemic toxicity.

## Discussion and conclusions

Nanotechnology for glioma therapy is booming. In previous studies, attentions have been focused on the specific ligand modified delivery system for glioma cells-targeting. Recently, the influence of the TME on cancer progression has become the focus (Yang & Gao, 2017). Thus, in order to improve prognostic and enhance anti-glioma efficacy, we developed a versatile drug delivery platform for glioma therapy with the regulation of TME.

By ion-pairing between positively charged DOX and negatively charged HA, DOX-HA-LPs successfully maintained the initial structure of HA without any chemical modification. Similar to the result of Yang et al. (2013), the nanoparticles which were established via ion-pairing might be a relative loose structure and instable in plasma (Figure S3). To solve the problem, these nanoparticles were encapsulated in liposomes (Park et al., 2015). DOX-HA-LPs achieved different release behavior with free DOX both *in vitro* (Figure 1(d)) and *in vivo* (Table S3). However, circulating behavior was not desired with a fast release in a short period of time after injection. This phenomenon might result from scavenging by mononuclear phagocyte system (MPS). Outer phospholipid membrane which was added for solving instability in plasma might shelter part of carboxylic and result in uptake by MPS (Figure S4) (Allen, 1994). To improve the circulating time of DOX-HA-LPs, we could attempt to optimize E80 to DSPE-mPEG2000 or add plasticizing agent to enhance the phospholipid membrane. What is more, we also could encapsulate DOX-HA-NPs in erythrocyte membrane (Hu et al., 2011). In our future research, improving circulating time of DOX-HA-LPs was an important work.

HA was proven to specifically bind to surface CD44 receptor. In our study, DOX-HA-LPs exhibited strong glioma site targeting potency (Figure 3) and anti-glioma efficacy (Figure 4). As the glioma grew and neovascularization formed, malignant glioma cells would secret soluble factors for degrading tight junctions, resulting in BBB permeability increasing and destroying (Yamada et al., 1982; Schneider et al., 2004). The high permeability of BBB helped DOX-HA-LPs invade into glioma. With EPR effect and CD44 receptor-mediated endocytosis, DOX-HA-LPs exhibited a higher glioma-bearing brains concentrated capability (Figure 3). However, compared with liver, glioma-bearing brain maintains a smaller size. Thus, as shown in Figure 3(b), it is able to understand that DOX-HA-LPs contribute to accumulation in liver more than that in glioma-bearing brain after 1 h. As aforementioned, TAMs play an important role in tumor development, angiogenesis, metastasis. DOX-HA-LPs selectively targeted TAMs and showed a potent anti-TAMs property *in vivo* (Figures 3(c) and 5). Moreover, DOX-HA-LPs exhibited anti-CSCs potency *in vivo* (Figure 5). Besides, CD44 was at the center of the signaling cascade, identified as a novel marker of vasculogenic tumor cells and exhibited an important role in the formation of VMs (Paulis et al., 2015). VMs, heavily related with CD44/c-Met signaling cascade, were inhibited (Figure 5). Although there was lower accumulation in brain compared with that in liver, DOX-HA-LPs which showed higher accumulation in glioma site compared with free DOX, would deplete TAMs, inhibit VMs, eliminate CSCs via CD44 mediated target ability, and further enhance anti-glioma efficacy (Figure 4) and significantly prolong survival time *in vivo* (Table S4).

In conclusion, HA modified, multi-functional liposomal nanoparticles have been identified as a comprehensive and safe anti-glioma DDS for overcoming multi-barriers of brain tumor and improving anti-tumor efficacy. The result suggested that DOX-HA-LPs extremely prolonged the survival time of glioma bearing mice. It offers two major advantages. First, HA is proven to be neither toxic nor immunogenic, and maintain initial structure in preparation method. Second, DOX-HA-LPs could achieve multiple purpose with depletion of TAMs and CSCs and inhibition of VMs. Although there was still problem about improving circulating time of DOX-HA-LPs which is needed to study further, HA modified liposomal nanoparticles represent a potentially attractive drug delivery platform for glioma treatment.

## Supplementary Material

Liuqing_et_al._Supplemental_Material.docx
