# Supplementary material for: Development a hyaluronic acid ion-pairing liposomal nanoparticle for enhancing anti-glioma efficacy by modulating glioma microenvironment
Source: Drug Deliv. 2018 Jan 29;25(1):388–97. doi: 10.1080/10717544.2018.1431979 (PMC6058578; doi:10.1080/10717544.2018.1431979)
Supplement: Liuqing_et_al._Supplemental_Material.docx [file IDRD_A_1431979_SM2140.docx]

Table S1

Characteristics of DOX-HA-LP (n=3，mean±SD)

| Entry | Size (nm) | PDI | Zeta (mV) | EE (%) |
| --- | --- | --- | --- | --- |
| DOX-HA-LP | 155.8±3.2 | 0.155±0.057 | -5.4±1.1 | 94.5±0.7 |

Table S2

*In vitro* cytotoxicities of DOX formulations against C6 and RAW264.7 cells after incubation for 24h

| Entry | IC_50_ (μg/mL) | | | |
| --- | --- | --- | --- | --- |
|  | Free DOX | DOX-HA-LP | DOX-HA^(-)^-LP | Blank liposomal nanoparticle |
| C6 | 111.52±0.34 | 6.57±0.41****^a^ | 13.95±0.19**^b^ | 232.25±37.47****^b^ |
| RAW264.7 | 5.73±0.67 | 0.04±0.007**^a^ | - | - |

Data represent mean ± SD (n = 5).

^a^ ** *p* < 0.01, **** *p* < 0.0001 vs Free DOX.

^b^ ** *p* < 0.01, **** *p* < 0.0001 vs DOX-HA-LP.

Table S3

Pharmacokinetic parameters of DOX formulations

| Sample | Free DOX | DOX-HA-LP |
| --- | --- | --- |
| Dose (mg/kg) | 5.0 | 5.0 |
| AUC_(0-∞)_ (μg/L*h) | 905.15 | 2270.62* |
| t_1/2_ (h) | 0.040 | 0.101* |

AUC, area under the curve. * *p* < 0.05

Table S4

Median (days) and ILS (%) of mice bearing C6 glioma treated with saline and different Dox formulations (n = 10).

| Groups | Median (days) | Mean survival time  (days) | ILS (%) | |
| --- | --- | --- | --- | --- |
|  |  |  | DOX | DOX-HA-LP |
| Saline | 21.5 | 23.7±2.7 | 37.1 | 97.1** |
| DOX | 31.0 | 32.5±4.6 | / | 45.9* |
| DOX-HA-LP | 53.0 | 46.7±6.5 | / | / |

ILS (increase in life span) = (T/C－1)×100%, where T and C represents the mean survival time (days) of the treated and control groups, respectively. *, *p* < 0.05. **, *p* < 0.01.

Table S5. Effect of molecular weight on the size and EE (%) of DOX-HA-NPs

| Molecular weight of HA (kDa) | Particle size (nm) | EE (%) |
| --- | --- | --- |
| 6 | 171.3±11.1 | 45.2 |
| 20 | 175.2±10.2 | 51.1 |
| 77 | 180.4±13.1 | 85.0 |
| 300 | 203.5±12.6 | 76.1 |
| 1000 | 222.9±14.6 | 74.4 |


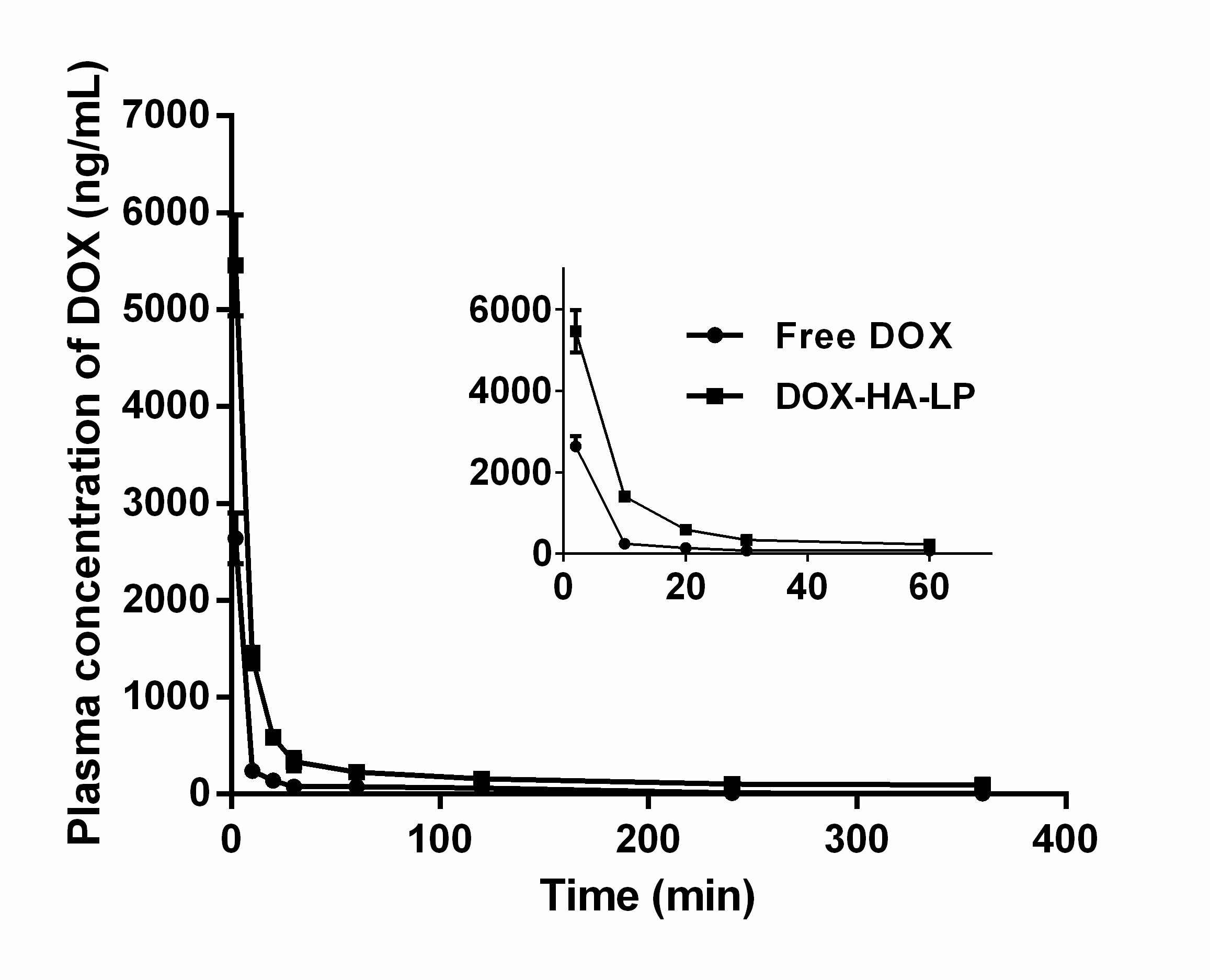


Figure S1

*In vivo* pharmacokinetic profiles after intravenous injection of DOX formulations in rats. Data represent mean ± SD. (n = 5)


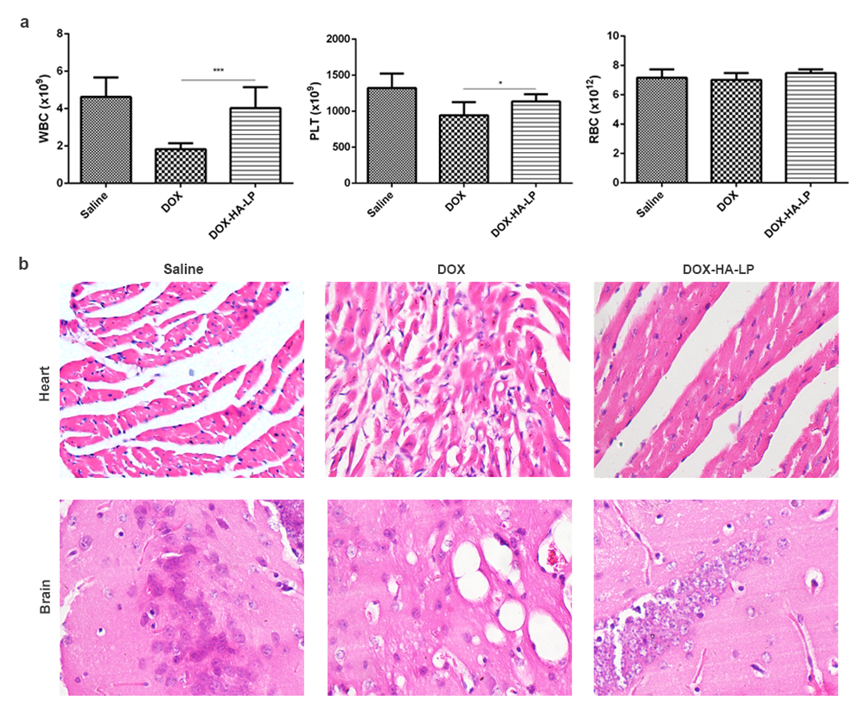


Figure S2

1. Leukocyte (WBC), erythrocyte (RBC) and platelets (PLT) cell counts of saline, free DOX and DOX-HA 3 days after treatment. Data represent mean ± SD (n = 7). * *p* < 0.05, *** *p* < 0.001.
2. Histological evaluation of hearts and brains from healthy mice after treatment with saline, free DOX and DOX-HA-LPs. Hearts were isolated and stained with hematoxylin and eosin (H&E). Scale bar, 200 μm.





Figure S3

The stability of DOX-HA-NP in plasma and PBS (pH 7.4) for 72 h. Data represent mean ± SD (n = 3).





Figure S4

Cellular uptake of DOX formulations after incubation with RAW264.7 cells for 4 h. Data represent mean ±SD (n = 3). * p < 0.05, ** p < 0.01.


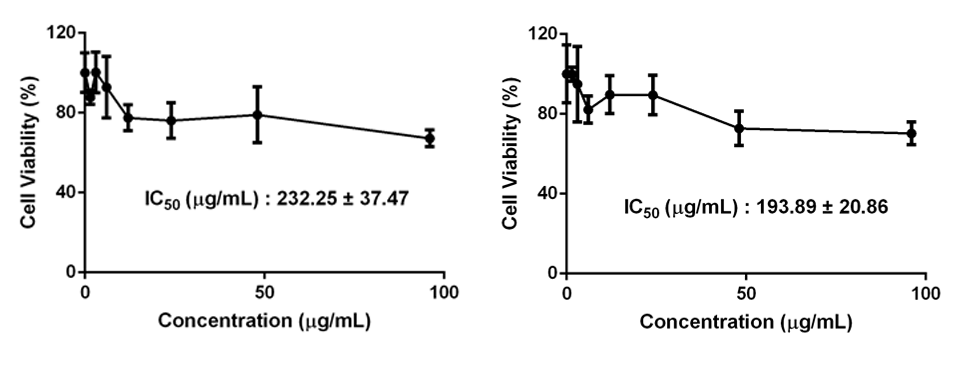


Figure S5.

Cell viability (%) and value of IC_50_ (μg/mL) after treatment with blank liposomal nanoparticles at 24 h.





Figure S6

*In vivo* pharmacokinetic profiles after intravenous injection of DOX formulations in rats. Data represent mean ± SD. (n = 5)





Figure S7

The mean concentrations (μg/g) of DOX in brain and liver tissues of C6 glioma-bearing mice after intravenous administration of DOX-HA-LPs at 0.5, 1, 2 and 4 h. Data represent mean ± S.D. (n = 5)
